# Manufacturing Shape-Controllable Flexible PEDOT/rGO Composite Electrodes for Planar Micro-Supercapacitors

**DOI:** 10.3390/ma17092144

**Published:** 2024-05-03

**Authors:** Haiwei Hu, Yanyan Guo, Jiang Zhao

**Affiliations:** College of Integrated Circuit Science and Engineering, Nanjing University of Posts and Telecommunications, Nanjing 210023, China; 17314416686@163.com

**Keywords:** flexibility, micro-supercapacitors, laser scribing, Poly(3,4-ethylenedioxythiophene), graphene oxide, composite electrode, areal capacitance

## Abstract

Flexible electronic products, with their characteristics of flexibility and wearability, have attracted significant attention and have become an important direction in the research and development of the electronics industry. Planar micro-supercapacitors (MSCs) with flexible composite electrodes can provide reliable energy support for these products, propelling their further development. The research employed a quick, effective, and environmentally friendly method of laser scribing to create shape-controllable flexible composite electrodes on composite films of Poly(3,4-ethylenedioxythiophene) and graphene oxide (PEDOT/GO), which were subsequently assembled into MSCs. An analysis of the composite electrode morphology, structure, and elemental distribution was conducted through the utilization of SEM, TEM, and XPS techniques. Following this, a comprehensive evaluation of the electrochemical performance of the flexible MSCs was carried out, which included cyclic voltammetry (CV), galvanostatic charge/discharge (GCD), and assessment of cyclic stability. The analysis of the CV results indicated that the MSCs achieved the areal capacitance of 5.78 mF/cm^2^ at 5 mV/s. After 5000 cycles at a current density of 0.05 mA/cm^2^, the capacitance retention rate was 85.4%. The high areal capacitance and strong cycle stability of MSCs highlight the potential of PEDOT/reduced graphene oxide (PEDOT/rGO) electrodes in electrode applications.

## 1. Introduction

Due to the depletion of fossil fuels and the exacerbation of environmental pollution [1], attention has shifted towards the development of renewable energy sources. However, the production capacity of renewable energies, such as wind and solar energy, is unstable and cannot be directly stored, existing only in the form of electricity [2]. This has accelerated the development of batteries and supercapacitors [3]. Supercapacitors, which are also referred to as electrochemical capacitors, are a modern form of energy storage that falls between conventional capacitors and batteries [4,5]. Supercapacitors are well known for their impressive power density [6,7], making them candidates for rapid power supply and charging [8]. Based on their charge storage mechanism, supercapacitors are categorized into electric double-layer capacitors (EDLCs) and pseudocapacitors [9,10]. Carbon materials, with their excellent conductivity, stable chemical properties, high specific surface area, low cost, and mature processing technology, are considered high-quality electrode materials for EDLCs [11,12]. The electrode materials for pseudocapacitors mainly include metal oxides and conductive polymers [13,14]. Pseudocapacitors store energy through reversible reactions on the surface of the electrode, demonstrating a mechanism that is more intricate than that of EDLCs [15,16,17,18].

In recent years, with the rise of new wearable electronic products, there has been an increasing interest in research on flexible planar micro-supercapacitors (MSCs) [19,20,21]. Graphene, due to its unique properties, has shown great potential as an electrode material for MSCs [22]. The mainstream fabrication techniques for graphene electrode materials include inkjet printing [23], laser scribing [24], and laser-induced approaches [25,26,27], with laser scribing being significantly more efficient than inkjet printing and comparable to laser-induced methods. Firstly, the laser scribing technique is lauded for its exceptional precision and high resolution, capable of achieving micrometer-level intricate patterns. This precision is crucial for applications requiring high-resolution patterns, such as the fabrication of graphene electrodes in supercapacitors, where exact electrode patterning can significantly impact device performance. In contrast, the preparation of graphene electrodes via inkjet printing requires the pre-processing of graphene powder to formulate the ink. Due to the hydrophobic nature of graphene, it is common to add sodium carboxymethyl cellulose (CMC) to enhance the stability of the dispersion. While CMC helps stabilize the ink, its addition can introduce impurities that may weaken the electrochemical performance of supercapacitors by influencing both the conductivity and the electrochemical reactivity of the electrode material. MSCs based on graphene electrode materials possess advantages such as short charging times, long cycle stability, and greater power density, holding tremendous application prospects in flexible micro-electronic devices [28,29,30,31]. Poly(3,4-ethylenedioxythiophene) (PEDOT), as a conductive polymer material, exhibits good conductivity, electrochemical stability, and reversible redox properties [32], making it suitable for use as an electrode material in supercapacitors [33,34,35,36]. It is noteworthy that most high-performance MSCs are currently based on composite electrode materials. The use of graphene alone as an electrode material for MSCs is limited by its lower electrochemical activity, affecting the electrode’s specific capacitance and reliability. Similarly, using PEDOT alone as an electrode material for MSCs is not ideal, as PEDOT nanoparticles have a lower specific surface area than graphene, meaning there are fewer active areas available for reactions between the electrode and the electrolyte, thus reducing the performance of the capacitor. Therefore, combining graphene and PEDOT into a composite electrode material is expected to overcome their individual limitations and improve the performance of MSCs, pushing the development and application of supercapacitor technology. Fuwei Liu [37] developed a supercapacitor utilizing the rGO/PEDOT/polyaniline composite electrode, which achieved a specific capacitance of 535 F/g and exhibited good cyclic stability. However, the fabrication process for the composite electrode is complex, making it unsuitable for direct use in flexible micro-supercapacitors (MSCs). In another study, Shiyuan Li [38] successfully fabricated an rGO/PEDOT composite electrode using a hydrothermal polymerization technique, which achieved a conductivity of 88.5 S/cm and a specific capacitance of 202.7 F/g. Nevertheless, the fabrication process for this electrode is challenging to control.

In this work, novel flexible PEDOT/rGO composite electrodes with controllable shapes were fabricated through an extremely simple and highly effective laser scribing technique. PEDOT not only provides additional electrochemical activity to enhance the performance of MSCs, but also improves the mechanical strength and flexibility of the electrodes, thereby increasing the reliability of MSCs. The high electrical conductivity of rGO facilitates rapid electron transport on the electrode surface, and its high specific surface area means that there are more active sites available for electrochemical reactions, further enhancing the electrochemical performance of MSCs. The electrochemical testing results of the flexible MSCs indicate that the maximum areal capacitance achieved by the PEDOT/rGO electrode-based MSCs is 5.78 mF/cm^2^ at 5 mV/s. Repeated charge/discharge tests conducted on the MSCs with optimal performance show that, under a current density of 0.05 mA/cm^2^, the retention of capacitance stands at 85.4% following 5000 cycles. All of these demonstrate that the fabricated flexible PEDOT/rGO composite electrodes hold application potential in electrodes for planar MSCs.

## 2. Experiment

### 2.1. Materials

The uniform, precipitate-free, single-layer graphene oxide (GO) dispersion with a concentration of 2 mg/mL, synthesized using an improved Hummers method, was obtained from Tanfeng Technology Co., Ltd., located in Suzhou, China. The Orgacon conductive polymer PEDOT:PSS dispersion, containing an active chemical content of PEDOT:PSS at 1.2% wt, was provided by Agfa-Gevaert N.V., Belgium. Polyvinyl alcohol (PVA) was obtained from Sinopharm Chemical Reagent Co., Ltd., Shanghai, China, and crystalline lithium chloride was sourced from Aladdin Biochemical Technology Co., Ltd., Shanghai, China.

### 2.2. Fabrication of PEDOT/rGO Electrodes

**Figure 1:**
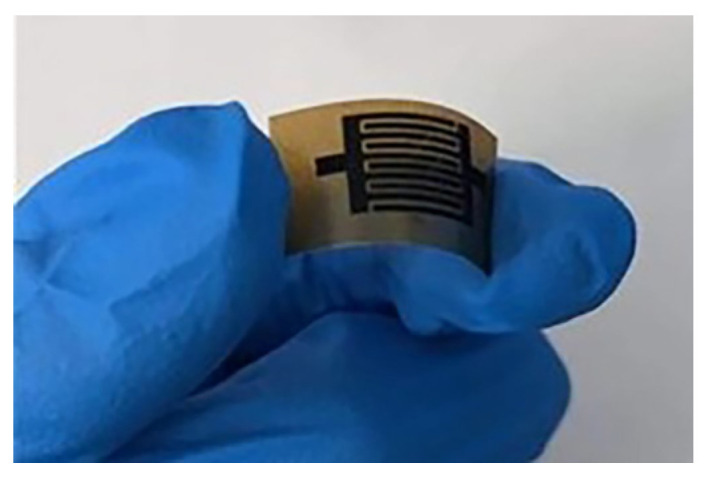
The physical sample of the PEDOT/rGO electrode.

First, 10 mL of GO dispersion was mixed with no more than 1 mL of PEDOT:PSS dispersion in a beaker, and the mixture was thoroughly stirred on a magnetic stirrer to ensure uniformity. Subsequently, a polyethylene terephthalate (PET) substrate with a diameter of 8 cm was prepared. The PEDOT/GO mixture was carefully drawn into a syringe and evenly dispensed onto the surface of the PET substrate. A coating rod was used to gently spread the mixture, ensuring the formation of a uniform and smooth layer. The mixture was then left to cure naturally at room temperature, forming a PEDOT/GO film. Finally, to pattern the PEDOT/GO film, a laser engraving machine with a wavelength of 405 nm and the maximum output power of 1.5 W was used. The laser power was set to 27.1%. Under these settings, the interdigital PEDOT/rGO electrode was fabricated within two minutes. Figure 1 presents a photograph of the fabricated PEDOT/rGO electrode sample.

### 2.3. Electrolyte

Here, 0.6 g of polyvinyl alcohol (PVA) powder was mixed with 5 mL of deionized water. Using a constant temperature magnetic stirrer, the mixture was steadily heated to 85 °C under vigorous stirring by a water bath method, and the stirring was continued until the mixture completely turned into a transparent gel. After cooling to room temperature, the transparent gel was further processed by adding 1 mL of a 0.6 g/mL LiCl solution. Upon thorough stirring, the PVA/LiCl gel electrolyte was obtained.

### 2.4. Construction of Planar MSCs

**Figure 2:**
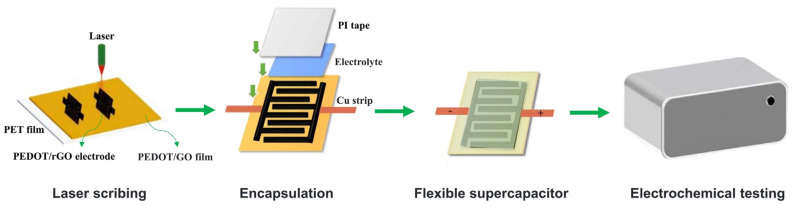
The encapsulation and testing processes of MSCs based on PEDOT/rGO electrodes.

The interdigital PEDOT/rGO electrode with a length of 10 mm, finger width of 0.7 mm, and inter-finger spacing of 0.4 mm was uniformly coated with the PVA/LiCl gel electrolyte. To enhance electrical conductivity, copper strips were added to both sides of the electrode. Additionally, a low-temperature conductive silver paste, curing at 80–150 °C, was applied at the interfaces between the copper strips and the electrode to ensure excellent electrical contact performance. Subsequently, the entire assembly was placed in an electrically heated, constant-temperature, air-circulating oven set at 80 °C to accelerate the curing process. Approximately twenty minutes later, the conductive silver paste had solidified. The solidification of the conductive silver paste secured the copper strips, ensuring the reliability and stability of the electrical connections. Finally, to complete the encapsulation process and protect the electrode, a layer of PI tape was carefully applied over the electrode that was coated with the electrolyte. After the encapsulation process, the dimensions of the micro-supercapacitor (MSC) were measured to be 13 mm in length and 8 mm in width. Figure 2 shows the sequential steps involved in the encapsulation process of MSC.

### 2.5. Characterization

The morphology of the electrodes was analyzed using field emission scanning electron microscopy (FESEM, Regulus 8100, Hitachi, Tokyo, Japan). The GO dispersion doped with PEDOT:PSS dispersion was analyzed using transmission electron microscopy (TEM, Talos F200X G2, Thermo, Waltham, MA, USA). Raman spectra were measured using a Raman spectrometer (Scientific DXR, Thermo, Waltham, MA, USA). The elemental composition was determined by X-ray photoelectron spectroscopy (XPS, Scientific K-Alpha, Thermo, Waltham, MA, USA).

### 2.6. Electrochemical Measurements

The CHI660E (Chenhua, Shanghai, China) system was used to test the electrochemical performance. Cycling performance tests were carried out on a BTS-3000 (Neware, Shenzhen, China). Analyses of electrochemical involved cyclic voltammetry (CV), galvanostatic charge/discharge (GCD), electrochemical impedance spectroscopy (EIS), and cyclic stability assessment. According to the CV curve, the areal capacitance at different scanning rates was calculated according to the equation *C_A_ = ∫IdV/S*Δ*Vv*, where *C_A_* (mF/cm^2^) denotes the areal capacitance, *∫IdV* signifies the integral under the CV curve, *S* denotes the area of supercapacitor, Δ*V* (V) represents the voltage window, and *v* (mV/s) indicates the scanning rate.

## 3. Results and Discussion

### 3.1. Surface Morphology Analysis

**Figure 3:**
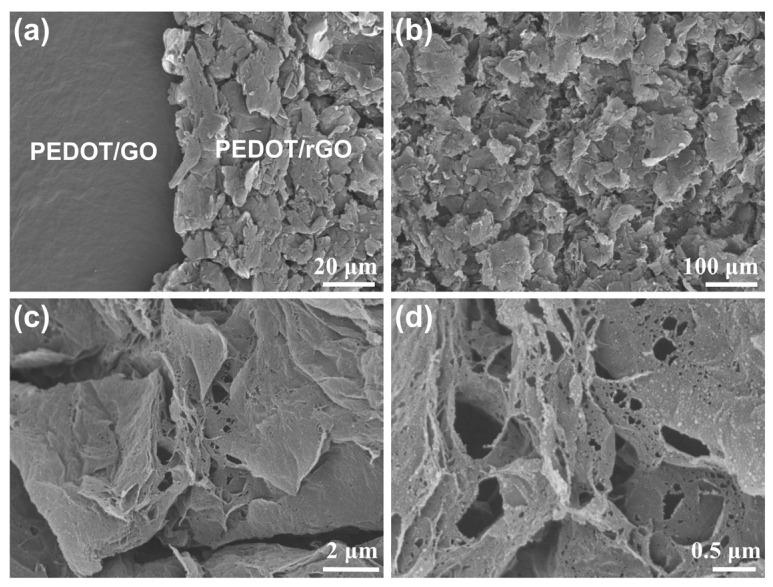
Analysis of material morphology: (**a**) the alteration in surface texture of the PEDOT/GO film due to laser scribing; (**b**) SEM image at low magnification; (**c**,**d**) SEM images at higher magnification.

The alterations in surface morphology of the PEDOT/rGO composite electrode due to laser scribing were analyzed through Scanning Electron Microscopy (SEM), illustrated in Figure 3a–d. Figure 3a presents the surface appearance of the PEDOT/GO composite material before laser treatment on the left, and the surface appearance of the PEDOT/rGO composite electrode on the right. This observation suggests that the laser scribing process successfully converts graphene oxide (GO) to reduced graphene oxide (rGO). Figure 3b demonstrates a rough surface of the composite electrode, showcasing a distinct layered and interconnected porous structure. Figure 3c depict the surface appearance of the composite electrode at increased magnifications, and the SEM image in Figure 3d displays a significant quantity of PEDOT nanoparticles adhering to the rGO framework.

**Figure 4:**
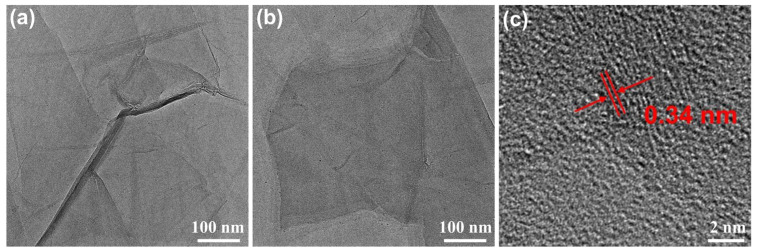
TEM images of the mixture of GO dispersion and PEDOT:PSS dispersion at different magnifications: (**a**,**b**) TEM images with a scale bar of 100 nm; (**c**) TEM image with a scale bar of 2 nm.

Figure 4 presents the TEM images of a mixture of GO dispersion and PEDOT:PSS dispersion. From Figure 4a, it can be observed that GO is highly electron transparent and exhibits a wrinkled shape. In Figure 4b, PEDOT nanoparticles are observed on the surface of GO. The high-resolution TEM (HRTEM) image in Figure 4c reveals that GO exhibits a few-layer structure of 2–3 layers, with an interlayer distance of about 0.34 nm, which is comparable to the interlayer distance of graphite.

### 3.2. Structure Analysis

**Figure 5:**
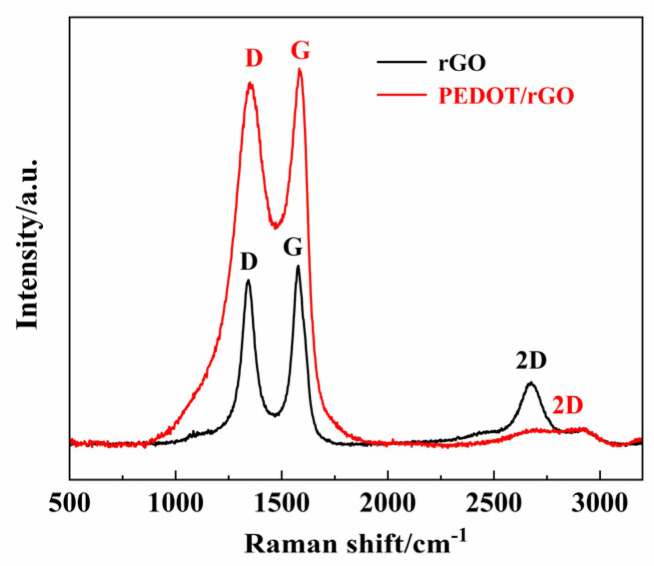
Raman spectra of rGO and PEDOT/rGO electrodes.

Figure 5 presents the Raman spectra of rGO and PEDOT/rGO electrode materials. The D peak, G peak, and 2D peak associated with rGO are located 1342 cm^−1^, 1578 cm^−1^, and 2650 cm^−1^, respectively (where the D and G peaks are the Raman characteristic peaks of carbon atom crystals, and the width of the 2D peak correlates with the uniformity of interlayer spacing and the degree of defects). For the PEDOT/rGO electrode materials, the D and G peaks appear at 1350 cm^−1^ and 1582 cm^−1^, indicating the presence of carbonaceous components within the composite electrode material [39]. Further analysis reveals that the intensity ratio of the D to G bands (ID/IG) for PEDOT/rGO is 0.97, which is higher than the 0.89 of rGO, suggesting that the presence of PEDOT nanoparticles promotes the formation of disordered multilayer graphene [40]. Additionally, a very broad 2D peak appears at 2700 cm^−1^ in the Raman spectra of PEDOT/rGO, reaffirming that the presence of PEDOT nanoparticles favors the production of disordered multilayer graphene [41].

**Figure 6:**
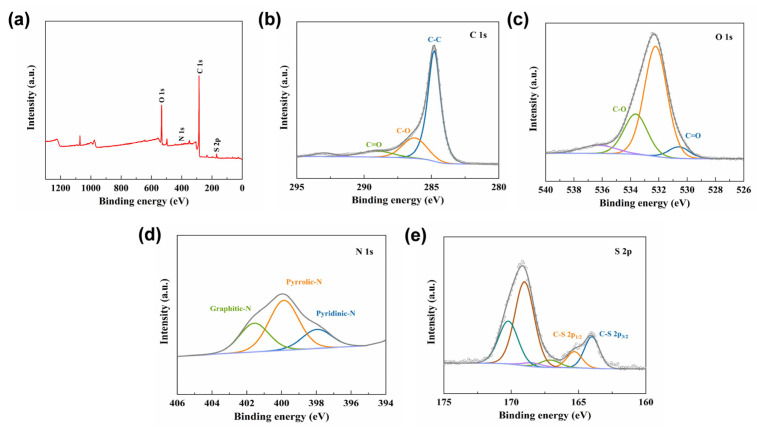
XPS spectra of the composite electrode: (**a**) full spectrum; (**b**) C 1s; (**c**) O 1s; (**d**) N 1s; (**e**) S 2p.

Figure 6 displays the XPS analysis of the PEDOT/rGO composite electrode material. The XPS technique reveals information on the chemical composition and chemical bonds of PEDOT/rGO. From the full XPS spectrum shown in Figure 6a, a significantly high peak of C 1s, along with lower peaks of O 1s, N 1s, and S 2p, can be observed. Figure 6b presents the high-resolution XPS spectrum of C 1s, showcasing three peaks located at 284.8, 286.3, and 289.1 eV, corresponding to the C−C, C−O, and C=O bonds [42]. Figure 6c, the high- resolution XPS spectrum of O 1s, exhibits two typical peaks corresponding to the C=O (530.6 eV) and C−O (533.6 eV) bonds [43]. The high-resolution XPS spectrum of N 1s shown in Figure 6d is fitted with three peaks at 397.9, 399.8, and 401.5 eV, attributed to pyridinic N, pyrrolic N, and graphitic N bonds [44]. Figure 6e shows the high-resolution XPS spectrum of S 2p, with the presence of C−S 2p3/2 (164.0 eV) and C−S 2p1/2 (165.3 eV) bonds, proving the formation of chemical bonds between sulfur and carbon atoms [45].

### 3.3. Electrochemical Performance Analysis

**Figure 7:**
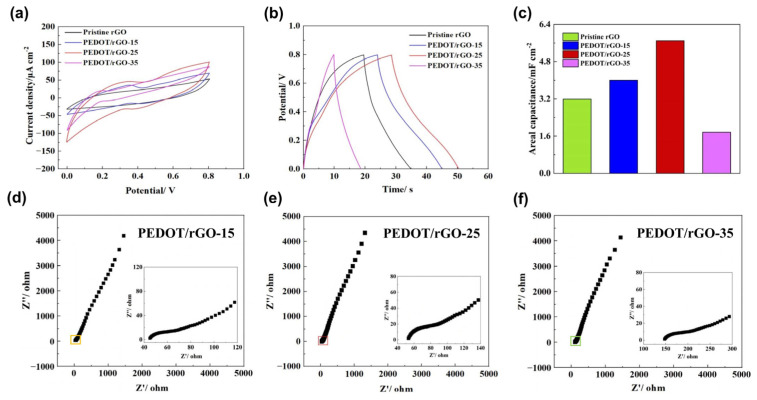
Electrochemical characteristics of the PEDOT/rGO MSC: (**a**) the CV curves at 5 mV/s; (**b**) the GCD curves at 70 μA/cm^2^; (**c**) the areal capacitance of MSCs; (**d**) the EIS graphs of the PEDOT/rGO-15 MSC; (**e**) the EIS graphs of the PEDOT/rGO-25 MSC; (**f**) the EIS graphs of the PEDOT/rGO-35 MSC.

Figure 7 demonstrates the study of the electrochemical performance of MSCs and the optimization of PEDOT/rGO composite electrodes, where the varying content of PEDOT directly affects the performance of individual PEDOT/rGO composite electrodes, with a specific ratio yielding optimal electrode performance. The fabrication of a reduced graphene oxide (rGO) electrode begins with its precursor, a GO film. After patterning the GO film using the laser scribing technique, the surface of the film is converted into an rGO electrode. For convenience, the MSC assembled based on this rGO electrode is designated as pristine rGO MSC. The fabrication of a PEDOT/rGO electrode begins with a composite precursor film. Initially, 10 mL of a GO dispersion with a concentration of 2 mg/mL is mixed with a specific volume of a PEDOT:PSS dispersion containing 1.2% wt active chemical content of PEDOT:PSS. After curing, the mixture forms a PEDOT/GO-V film, where ‘V’ denotes 100 times the volume of the PEDOT:PSS dispersion in the mixture. Subsequently, the PEDOT/GO-V film is precisely patterned into the desired electrode shape via laser scribing technique, thereby resulting in the formation of a composite. Finally, this composite electrode is assembled into a PEDOT/rGO-V MSC. Figure 7a presents the CV curves of the MSCs. For pristine rGO MSC, the shuttle-like contour of the CV curve indicates that the areal capacitance of pristine rGO MSC is solely provided by the electric double-layer capacitance (EDLC). In contrast, for PEDOT/rGO-V MSC, the fluctuating CV curves reveal that the areal capacitance of PEDOT/rGO-V MSC is provided by both EDLC and pseudocapacitance. Additionally, the integral area of the CV curves increases from pristine rGO MSC to PEDOT/rGO-25 MSC, and then decreases from PEDOT/rGO-25 MSC to PEDOT/rGO-35 MSC. Figure 7b illustrates the GCD curves of the MSCs, which show that the discharge time of PEDOT/rGO-V MSC follows a similar trend of initial increase followed by a decrease, aligning with the changes observed in the integral area of the CV curves. The areal capacitance of the MSCs shown in Figure 7c are calculated based on the CV curves presented in Figure 7a. After uniformly mixing 10 mL of GO dispersion with 0.25 mL of PEDOT:PSS dispersion, a PEDOT/GO-25 film is formed, which contains 20 mg of GO and 3 mg of PEDOT. Based on this specific composition of precursor film, a composite electrode is subsequently fabricated and assembled into a PEDOT/rGO-25 MSC. According to Figure 7c, the PEDOT/rGO-25 MSC exhibits the highest areal capacitance, indicating that the composite electrode made from the PEDOT/GO-25 film exhibits optimal performance. This further suggests that even slight variations in the PEDOT content of the composite precursor film can significantly impact the electrochemical performance of the electrode material. Figure 7d–f display the electrochemical impedance spectroscopy (EIS) graphs for PEDOT/rGO-15, PEDOT/rGO-25, and PEDOT/rGO-35, respectively. Understanding the mass content of PEDOT and GO in the composite precursor film is beneficial for EIS analysis. After mixing 10 mL of GO dispersion with 0.35 mL of PEDOT:PSS dispersion, a PEDOT/GO-35 film is formed, which contains 20 mg of GO and 4.2 mg of PEDOT. Based on this specific composition of precursor film, a composite electrode is subsequently fabricated and assembled into a PEDOT/rGO-35 MSC. In the high-frequency domain, the x-coordinate at which the impedance curve intersects the real axis represents the equivalent series resistance (ESR) of the MSC, incorporating the resistances of both the electrode and the electrolyte. According to the insets in the EIS graphs, which provide a magnified view of this domain, the ESR values are 45.7 Ω for PEDOT/rGO-15 MSC, 52.8 Ω for PEDOT/rGO-25 MSC, and 146.5 Ω for PEDOT/rGO-35 MSC. Analysis of the three EIS graphs reveals that the intercept on the real axis, which represents the ESR of the MSC, progressively increases as the concentration of PEDOT in the precursor film rises. Thus, with the electrolyte held constant, it is evident that the resistance of the electrode increases as the PEDOT concentration rises. Figure 7d–f demonstrate that the resistance of the electrode fabricated from the PEDOT/GO-35 film is significantly higher than those prepared from the other two precursor films. While the variation in ESR between PEDOT/rGO-15 MSC and PEDOT/rGO-25 MSC is slight, a significant increase observed between PEDOT/rGO-25 MSC and PEDOT/rGO-35 MSC clearly demonstrates the impact of higher PEDOT concentrations on the performance of PEDOT/rGO composite materials. The porous structure of rGO in these materials typically enhances ion and electron transport, thereby improving overall conductivity. However, the impact of PEDOT concentration is critical: a moderate concentration of PEDOT slightly increases the resistance, while excessively high concentrations significantly increase it, leading to detrimental effects. Specifically, high concentrations result in the clogging of rGO pores and the accumulation of polymer chains, which impede both ion and electron mobility within the composite material, thereby reducing its conductivity. The reduced conductivity leads to uneven charge distribution across the electrode material, which restricts effective charge storage in specific areas. Consequently, this condition causes wastage of active electrode material and decreases the areal capacitance. If the detrimental effects of reduced conductivity, caused by an excessive concentration of PEDOT nanoparticles in the electrode material, outweigh the benefits of enhanced electrochemical activity, the areal capacitance of the PEDOT/rGO-35 MSC will be lower compared to that of the pristine rGO MSC.

**Figure 8:**
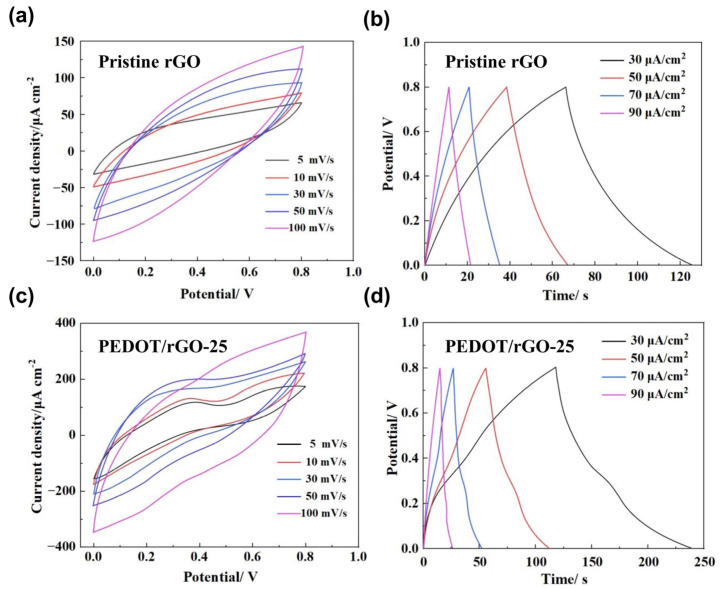
Electrochemical performance tests: (**a**) CV curves of the pristine rGO MSC at 5–100 mV/s; (**b**) GCD curves of the pristine rGO MSC at 30–90 μA/cm^2^; (**c**) CV curves of the PEDOT/rGO-25 MSC at 5–100 mV/s; (**d**) GCD curves of the PEDOT/rGO-25 MSC at 30–90 μA/cm^2^.

Figure 8 presents the results of CV and GCD tests for pristine rGO MSC and PEDOT/rGO-25 MSC. Figure 8a shows the CV curves of pristine rGO MSC, where the curve at 5 mV/s exhibits a shuttle-like contour. This indicates that pristine rGO MSC is capable of accumulating and releasing charge without any chemical reaction, relying solely on electric double-layer capacitance (EDLC) for the storage and release of energy. The EDLC arises from the electrostatic accumulation of electrolyte ions at the graphene electrode surface in pristine rGO MSC. Based on the CV curve at 5 mV/s, the areal capacitance of pristine rGO MSC is calculated to be 3.19 mF/cm^2^. Figure 8b shows that the GCD curves of pristine rGO MSC at 30–90 μA/cm^2^ form approximately isosceles triangles, indicating that pristine rGO MSC, fabricated from GO film, exhibits good electrode reversibility and capacitive characteristics. Figure 8c displays the CV curves of PEDOT/rGO-25 MSC at 5–100 mV/s, where noticeable redox peaks are observed. This finding indicates that the PEDOT/rGO-25 MSC stores energy through a combination of EDLC and pseudocapacitance. The pseudocapacitance in the PEDOT/rGO-25 MSC originates from rapid and reversible Faradaic processes associated with PEDOT. These processes involve the transfer of electrons and ions at or near the surface of the electrode material, thereby facilitating the efficient storage and release of energy. In the CV testing of the PEDOT/rGO-25 MSC, even as scan rates increased, the displacement of the redox peaks on the CV curves remained very slight. This observation indicates that the electrode material can rapidly facilitate charge transfer reactions even under fast scanning conditions, while the ionic diffusion within the electrolyte is sufficiently swift to promptly adjust to changes in potential. This rapid responsiveness confirms the high reversibility of the electrode reactions across a range of scanning rates, thereby demonstrating excellent electrochemical kinetics at the electrode/electrolyte interface. The areal capacitance of the PEDOT/rGO-25 MSC is calculated to be 5.78 mF/cm^2^ based on the CV curve at 5 mV/s, meaning that PEDOT/rGO-25 MSC has achieved an 81% performance improvement compared to pristine rGO MSC. Figure 8d illustrates the GCD curves of the PEDOT/rGO-25 MSC at current densities ranging from 30 to 90 μA/cm^2^. Notably, a plateau observed during both the charging and discharging phases corresponds to the oxidation and reduction peaks identified in the CV curves. Furthermore, as the current density increases, the discharge time essentially halves, indicating that even under higher loads, PEDOT/rGO-25 MSC can respond quickly, demonstrating excellent rate performance.

**Figure 9:**
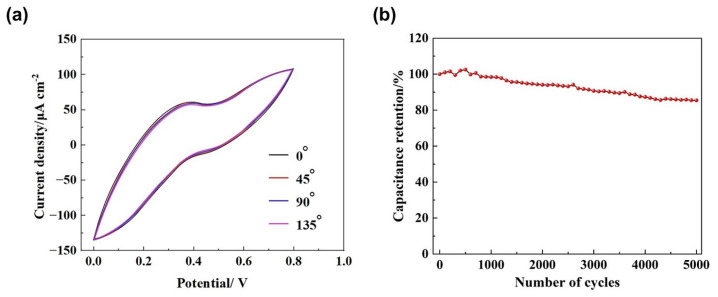
Flexibility and cyclic stability testing of the PEDOT/rGO-25 MSC: (**a**) CV curves obtained at various bending angles including 135 degrees, 90 degrees, 45 degrees, and 0 degrees; (**b**) the cyclic life curve.

Figure 9 illustrates the flexibility and cyclic stability tests of the PEDOT/rGO-25 MSC. Flexibility testing is performed by bending the MSC to various angles, while CV testing is employed to assess the electrochemical performance of the MSC under these different bending conditions. This method is used to evaluate the stability and reliability of its performance when subjected to changes in physical form. Specifically, CV measurements were performed on the MSC bent at various angles, including 135°, 90° (forming a right angle), 45°, and 0° (flat), with all tests conducted at scan rates of 5 mV/s. The flexibility of the MSC is assessed by comparing the integrated areas of the CV curves obtained at different bending angles. From Figure 9a, it is evident that the four CV curves closely overlap, demonstrating that the PEDOT/rGO-25 MSC exhibits nearly identical performance across all bending angles. This straightforward testing method confirms the robust flexibility of the fabricated PEDOT/rGO-25 MSC. Figure 9b illustrates the cyclic life curve of the PEDOT/rGO-25 MSC following 5000 charge/discharge cycles at a current density of 0.05 mA/cm^2^. An analysis of the curve reveals that the capacitance retention is 85.4%; this high level of capacitance maintenance suggests that the PEDOT/rGO-25 MSC exhibits excellent durability and stability.

Table 1 lists three types of previously reported MSCs, including the types of electrodes and electrolytes used, along with their respective areal capacitances. The data clearly demonstrate that MSCs utilizing PEDOT/rGO composite electrodes exhibit a certain leading edge in performance.

## 4. Conclusions

The energy storage mechanism of PEDOT/rGO MSCs is a composite mechanism, relying on both pseudocapacitance and electric double-layer capacitance (EDLC) for efficient energy storage. The pseudocapacitance originates from the rapid and reversible Faradaic processes of PEDOT, involving the transfer of electrons and ions in the surface or near-surface region of the electrode material, thus enabling the storage and release of energy. The conductive polymer framework of PEDOT provides a wealth of active sites and excellent conductivity, allowing it to exhibit high pseudocapacitive performance in electrochemical reactions. On the other hand, EDLC is generated by charge separation in the rGO layers, where charges form electrostatic attractions between the electrode surface and the electrolyte. This mechanism does not involve the chemical transformation of charges, making it a non-Faradaic process. By combining these two mechanisms, PEDOT/rGO MSCs not only achieve high energy storage, but also maintain good cycle stability and rapid charge–discharge capability. The preparation strategy of this composite material, by optimizing the interaction and ratio between PEDOT and rGO, further enhances its electrochemical performance, thereby expanding the application scenarios of flexible MSCs based on PEDOT/rGO composite electrodes.

## Figures and Tables

**Table 1:** Performance comparison of the PEDOT/rGO MSCs with other graphene-based MSCs.

Electrode	Electrolyte	Areal Capacitance (mF/cm^2^)	Ref.
NiOOH/Ni(OH)_2_/Graphene	PVA/KOH	0.75	[46]
rGO/CNT	PVA/H_3_PO_4_	2.6	[47]
Graphene/MWNT	PVA/H_3_PO_4_	2.54	[48]
PEDOT/rGO	PVA/LiCl	5.78	This work

## Data Availability

Data are contained within the article.
